# Effects of sleep-disordered breathing on serum lipid levels in children:a case control study

**DOI:** 10.1186/s12887-024-04577-6

**Published:** 2024-04-01

**Authors:** Lei Lei, XiaoYun Zhang, Binbin Wang, Fei Lei, Li Dai, Xiaoru Sun, Yu Zhao, Ping Zhu, Jian Zou

**Affiliations:** 1 Department of Otorhinolaryngology, Head&Neck Surgery, West China Hospital, West China Medical School, Sichuan University, Sichuan, China; 2 Department of Sleep Medical Center, West China Hospital, West China Medical School, Sichuan University, Sichuan, China; 3 West China Medical School, Sichuan University, Sichuan, China; 4 Department of Clinical Research Management, West China Hospital, Sichuan University, Sichuan, China

**Keywords:** Sleep-disordered breathing, Serum lipid, Obese, Children, Primary snoring, Obstructive sleep apnea syndrome

## Abstract

**Background:**

Sleep-disordered breathing (SDB) during childhood is common and includes a range of breathing abnormalities that range from primary snoring (PS) to obstructive sleep apnea syndrome (OSAS).Studies have shown that not only OSAS, but also PS, which is originally considered harmless, could cause cardiovascular, cognitive, behavioral, and psychosocial problems. Many researches are focused on the relation of OSA and serum lipid levels. However, little studies are focused on PS and serum lipid levels in children.We evaluated whether serum lipid (total cholesterol (TC), triglyceride (TG), high-density lipoprotein cholesterol (HDL-C),low-density lipoprotein cholesterol (LDL-C)) concentrations were associated with specific components of SDB, including indices of oxygen reduction index, lowest oxygen saturation, mean oxygen saturation. And we explored whether serum lipid levels were associated with different degree sleep disordered (PS and OSA group) and obese.

**Methods:**

This was a cross-sectional study. Children who were complained by their guardians with habitual snoring and(or) mouth breathing were collected in the SDB group. Normal children without sleep problem were matched in the control group. Subjects in the SDB group underwent polysomnography. The serum lipid profiles of all the children included TC, TG, HDL-C and LDL-C concentrations were measured by appropriate enzymatic assays.

**Results:**

A total of 241 with Apnea/Hypopnea Index ≥ 5 (AHI) were assigned to the OSAS group and the remaining 155 with normal AHI were assigned to the PS group. The values of TC, TG, LDL-C and LDL/HDL were significantly higher in the OSAS group than in the PS group, and the values in the PS group were significantly higher than the control group. Multiple regression analysis revealed serum TG only correlated negatively with lowest oxygen saturation. Body mass index-z score has a positive effect on TG in all the 1310 children (*P* = 0.031) and in SDB 396 children(*P* = 0.012). The level of serum TG in obese group was significantly higher than that in non-obese group.

**Conclusions:**

SDB had a very obvious effect on blood lipids, whereas PS without apnea and hypoxia. Obese only affects the aggregation of TG.

**Trial registration:**

ChiCTR1900026807(2019.10.23).

## Background

Sleep-disordered breathing (SDB) during childhood is common and includes a range of breathing abnormalities that range from primary snoring (PS) to obstructive sleep apnea syndrome (OSAS).PS is also referred to as simple or non-apneic snoring, and is regarded as the mildest form of SDB that does not have severe medical consequences. The most severe manifestation of SDB is OSAS, which is characterized by repeated episodes of upper-airway obstruction during sleep [1].

SDB is increasingly recognized as contributing to a substantive public health burden in both adults and children. Studies in pediatric populations have shown an association between SDB and alterations in metabolic syndrome, systemic blood pressure levels, echocardiographic findings and insulin resistance [2–4].These alterations seem to occur primarily in children with moderate to severe OSAS. Studies over the years have shown that not only OSAS, but also PS, which was originally considered harmless, can cause cardiovascular, cognitive, behavioral, and psychosocial problems [5, 6].Research on SDB, particularly in relation to PS and serum lipid levels in children, is sparse.

In this report, we reported the difference in serum lipid levels between normal children and children with SDB. We also quantified the association between serum lipid level and of children who had undergone standardized assessment of SDB by PSG. Using the AHI score, children with SDB were divided into the PS and OSA group. We addressed the hypothesis that serum lipid level was more pronounced among children with SDB than in those without SDB. In addition, we evaluated whether the association between serum lipid and SDB was independent of sex, age and course whether serum lipid was associated with specific components of SDB, including indices of oxygen reduction index, lowest oxygen saturation, mean oxygen saturation. Finally, we explored whether individual measures of serum lipid were associated with SDB independently of obese (Body mass index-z score, BMI-z score).

## Methods

### Subjects and physical assessment

The cross-sectional study: between December 2019 and December 2022, children who were complained by their guardians with SDB (habitual snoring and(or) mouth breathing more than 6 months) were collected in the SDB group. Normal children without snoring and mouths breathing at the same time were matched in the control group. Children were excluded from the study if they had an intercurrent upper respiratory tract infection or systemic infection within 4 weeks of recruitment, a history of tonsillectomy and(or) adenoidectomy, neuromuscular disease, craniofacial syndromes, cerebral palsy, sickle cell disease, mucopolysaccharide storage disease, immunodeficiency and mental or physical impairment.

The weight and standing height of the subjects were measured with a calibrated weighing scale and stadiometer according to standard anthropometric methods. Body mass index(BMI) was calculated as weight/height^2^ (kg/m^2^), and the z -score was derived using CDC reference data [7]. Children were defined as obese if their BMI -z score was greater than 1.645 [8], relative to age and gender.

### Polysomnography

Subjects in the SDB group underwent polysomnography, and performed using with an ambulatory polygraphic device. Obstructive apnea was defined as the absence of oronasal airflow for at least 2 breaths during. Central apnea was defined as the absence of oronasal airflow for at least 2 breaths during associated with an absence of inspiratory effort. Hypopnea was defined as at least a 50% reduction in oronasal airflow for at least 2 breaths during associated with at least a 3% decrease in oxygen saturation. The AHI was calculated as the mean number of apneas and hypopneas per hour of sleep. The obstructive and central apnea indexes were calculated as the mean number of obstructive and central apneas per hour of sleep, respectively. Subjects with an AHI score of > 5 score were designated as normal. AHI, oxygen reduction index (ORI), lowest oxygen saturation(L-SpO2), and mean oxygen saturation(M-SpO2) were recorded.

### Serum lipid measurement

Venous blood samples of experimental group were collected in the overnight fasting state after polysomnography. Blood samples of control group were collected on the morning of physical examination day after an overnight fast of at least 8 h. The lipid profile included total cholesterol (TC), triglyceride (TG), high-density lipoprotein cholesterol (HDL-C), and low-density lipoprotein cholesterol (LDL-C) concentrations, which were measured by appropriate enzymatic assays.

The study was approved by the Biomedical Research Ethics Committee of the West China Hospital of Sichuan University. Written informed consent was obtained from each patient before the study.

### Statistical analysis

Continuous data are expressed as mean ± SD. Continuous data between the groups were compared using the unpaired t test or the Mann-Whitney U test. Categorical data between the groups were compared using the Fisher exact test or the χ2 test. Spearman rank correlations were used to examine correlations between the continuous variables. Univariate and multiple regression analyses were performed to determine variables associated with TC, LDL-C, HDL-C and LDL-C/HDL-C. Because body mass index z-score (BMI-z), TG, AHI, ORI, L-SpO2, M-SpO2 were not normally distributed, logarithmically transformed values of these variables were used in the analyses. Multiple regression analysis was performed using explanatory variables that showed *P* < 0.05 on univariate analysis. A *P* < 0.05 was considered to be statistically significant. All analyses were performed using SPSS software (version 22.0; IBM SPSS, Armonk, NY, USA).

## Results

### Patient characteristics and overnight polysomnographic findings

A total of 1310 children were concluded in the study. In the SDB group, a total of 396 subjects with breathing problems having PSG results were recorded. And 241 with AHI>5 were assigned to the OSAS group and the remaining 155 with AHI ≤ 5 were assigned to the PS group. Nine hundred and fourteen normal children without sleeping problems during the same period were collected in the control group. Patient characteristics and overnight polysomnographic results were shown in Table 1.

**Table 1:** Patient characteristics and overnight polysomnographic results

	Control (*n* = 914)	PS (*n* = 155)	OSA (*n* = 241)	*P* value
Age, yrs	4 (3–7)	6 (4–7)	5 (4–7)	0.141
Male, n(%)	537 (58.8)	95 (61.3)	169 (70.1)	**0.006**
BMI-Z	-0.19 (-0.78 to 0.56)	-0.21 (-0.94 to 0.53)	-0.17 (-0.88 to 0.98)	0.408
BMI-Z > 1.65, n(%)	85 (9.3)	17 (11.0)	40 (16.6)	**0.005**
Course of disease	--	24 (12–36)	24 (12–36)	0.297
AHI	--	2 (1.1–3.2)	12.3 (7.05–20.7)	< 0.001
Oxygen reduction index	--	4.4 (2.58–7.03)	15.3 (8.15–24.7)	< 0.001
Lowest oxygen saturation	--	78 (67.75-85)	75 (66-82.75)	0.019
Mean oxygen saturation	--	96 (94–97)	95 (93–96)	0.040
Microarousal index	--	7.3 (5-9.7)	10.9 (8.05–14.65)	< 0.001

From the Table 1, boys and obese children were significantly more in OSA group than in PS group, and more common in the PS group than in the control group. No statistical difference was shown in age among the three groups. Course of disease between PS and OSAS groups have no difference.

### SDB leading to abnormal serum lipid parameters

SDB group were divided into the OSAS group and the PS group. From Table 2, we could see that the values of TC, TG, LDL-C and LDL/HDL were significantly higher in the OSAS group than in the PS group, and the values in the PS group were significantly higher than the control group. While the HDL-C in the OSAS group was significantly lower than that in the PS group, and this in the PS group was significantly lower than that in the normal group.

**Table 2:** TC, TG, LDL-C, HDL-C and LDL-C/HDL-C in the OSAS, PS and control group

	Control (*n* = 914)	PS (*n* = 155)	OSA (*n* = 241)	*P* value
TC	3.98 (3.58–4.49)	4.10 (3.75–4.57)	4.20 (3.67–4.63)	**0.005**
TG	0.73 (0.56–1.01)	0.76 (0.60-1.00)	0.80 (0.62-1.00)	**0.044**
LDL-C	2.15 (1.77–2.56)	2.24 (1.89–2.65)	2.32 (1.85–2.75)	**0.004**
HDL-C	1.56 (1.33–1.82)	1.54 (1.30–1.74)	1.48 (1.28–1.73)	**0.016**
LDL-C/HDL-C	1.38 (1.10–1.74)	1.41 (1.21–1.85)	1.52 (1.23–1.93)	**< 0.001**

Multiple regression analysis revealed that after adjusting age, gender, course of disease and BMI-z score, serum TG only correlated negatively with L-SpO2. Except that the lowest oxygen saturation had negatively effect on TG, night oxygen saturation (oxygen desaturation index, L-SpO2, M-SpO2) have no effects on other blood lipid parameters (TC, LDL-C, HDL-C and LDL-C/HDL-C). (Table 3)

**Table 3:** Multiple regression analysis the relationship between blood lipid parameters and overnight polysomnographic results

	TC		TG		LDLC		HDLC		LDLC/HDLC	
	β (95% CI)	*P*	β (95% CI)	*P*	β (95% CI)	*P*	β (95% CI)	*P*	β (95% CI)	*P*
AHI	-0.001 (-0.017 to 0.018)	0.968	0.005 (-0.003 to 0.013)	0.234	-0.001 (-0.015 to 0.013)	0.918	0.001 (-0.007 to 0.007)	0.914	-0.002 (-0.013 to 0.010)	0.798
Oxygen reduction index	-0.003 (-0.020 to 0.013)	0.681	-0.007 (-0.014 to 0.001)	0.069	0.001 (-0.012 to 0.014)	0.909	-0.002 (-0.008 to 0.005)	0.638	0.004 (-0.007 to 0.015)	0.489
Mean oxygen saturation	-0.004 (-0.017 to 0.008)	0.501	-0.002 (-0.008 to 0.004)	0.518	-0.003 (-0.013 to 0.008)	0.584	-0.001 (-0.007 to 0.004)	0.598	0.001 (-0.008 to 0.009)	0.892
Lowest oxygen saturation	-0.002 (-0.009 to 0.006)	0.636	-0.005 (-0.008 to -0.001)	**0.006**	0.001 (-0.006 to 0.006)	0.989	0.001 (-0.003 to 0.003)	0.835	0.001 (-0.005 to 0.006)	0.868
Microarousal index	0.016 (-0.004 to 0.035)	0.117	0.010 (0.001 to 0.019)	**0.026**	0.010 (-0.006 to 0.026)	0.238	0.001 (-0.008 to 0.007)	0.908	0.010 (-0.004 to 0.023)	0.154

### Obesity leads to abnormal serum TG metabolism

We adjusted for factors such as age, course of disease and gender, and found that there was still a close relationship between BMI-Z and TG in all children(*N* = 1310) and SDB children(*n* = 396). BMI-z score has a positive effect on TG in all the 1310 children (*P* = 0.031) and in SDB 396 children(*P* < 0.001).

We divided all the subjects and SDB groups into obese and non-obese groups. The LDLC/HDLC of obese children was significantly higher than that of non-obese children of SDB children. The HDLC of obese children was significantly lower than that of non-obese children of SDB children. The level of serum TG in obese group was significantly higher than that in non-obese group, regardless of whether it was the entire study population or children with SDB (Table 4). TC, LDL-C and LDL-C/HDL-C in SDB group was much higher than that in the control group (Table 5).

**Table 4:** TC, TG, LDL-C, HDL-C and LDL-C/HDL-C in obese and non-obese group

	Total Children (*n* = 1310)			SBD Children (*n* = 396)		
	BMI-Z ≤ 1.65 (*n* = 1168)	BMI-Z > 1.65 (*n* = 142)	*P* value	BMI-Z ≤ 1.65 (*n* = 339)	BMI-Z > 1.65 (*n* = 57)	*P* value
TC	4.01 (3.60–4.54)	4.15 (3.59–4.60)	0.289	4.18 (3.70–4.61)	4.12 (3.65–4.59)	0.674
TG	0.73 (0.57–0.99)	0.87 (0.59–1.14)	**0.002**	0.76 (0.61–0.98)	0.93 (0.70–1.17)	**0.004**
LDL-C	2.18 (1.81–2.59)	2.32 (1.82–2.61)	0.286	2.27 (1.87–2.71)	2.33 (1.98–2.70)	0.657
HDL-C	1.55 (1.33–1.79)	1.46 (1.23–1.78)	0.074	1.53 (1.33–1.75)	1.33 (1.18–1.60)	**< 0.001**
LDL-C/HDL-C	1.41 (1.13–1.77)	1.49 (1.16–1.96)	0.078	1.45 (1.21–1.86)	1.66(1.36–2.08)	**0.006**
Dyslipidemia	219 (18.8)	32 (22.5)	0.279	61 (18.0)	16 (28.1)	**0.075**

**Table 5:** TC, TG, LDL-C, HDL-C and LDL-C/HDL-C in non-obese group between SDB and control group

	Control (*n* = 829)	SDB (*n* = 339)	*P* value
TC	3.96(3.58–4.48)	4.19 (3.71–4.63)	**0.001**
TG	0.72 (0.56–1.11)	0.82 (0.69–1.17)	0.057
LDL-C	2.14 (1.73–2.60)	2.33(1.98–2.76)	**0.003**
HDL-C	1.58(1.31–1.89)	1.33(1.18–1.60)	0.184
LDL-C/HDL-C	1.34(1.13–1.80)	1.66(1.36–2.09)	**0.002**
Dyslipidemia	158 (19.1)	61 (18.0)	0.141

## Discussion

The main findings of the present study were as follows: (1) serum LDL-C, TG, TC and LDL-C/HDL-C were much higher in OSA and PS group than control group;(2) serum HDL-C were much lower in OSA and PS group than control group;(3) multiple regression analysis showed that nocturnal oxygen saturation (L-SpO2, M-SpO2, ORI) and was not associated with TG, LDL-C, HDL-C, LDL-C/HDL-C expect TG;(4) serum TG correlated negatively with L-SpO2 and positively with BMI-z and obesity.

Sleep disturbance in children can cause dyslipidemia, which has been confirmed in many previous studies [9]. Redline et al.studied a group of teens and showed that adolescents with sleep respiratory disorders have seven times more chance of having metabolic alterations than adolescents with no sleep disorders [3]. We found that children with sleep disorder had worse blood lipid indicators than children without sleep disorder. Even in the PS group, which is considered as harmless [5], it is also found that there is obvious blood lipid abnormality compared with the normal group. The first interesting finding of Lorenzo Loffredo s study is that endothelial dysfunction, high oxidative stress and NOX^2^ activation characterized not only in children with serious SDB, such as those with OSA, but also in children with PS [10]. In our three subgroups of OSAS group, serum lipid indicators were not related to the degree of AHI. According to the above analysis and our results, children with PS do not experience intermittent hypoxia. Therefore, we speculate that there may be other important reasons but L-SpO2 for abnormal blood lipid indicators in children caused by SDB. In fact, the review study of Sun also found the pathogenetic link of SDB with dyslipidemia remains obscure [11]. It is also possible that the reason why SDB causes lipid metabolism changes in children is may not hypoxia, but a combination of many reasons like abnormal sleep structure and shorter sleep during time leading to the change of endothelial dysfunction, high oxidative stress, NOX2 activation and other metabolic mechanism. With regard to cross-sectional findings, one study reported that longer sleep duration as measured objectively with actigraphy had a beneficial effect on overall lipid metabolism in a community cohort of children aged 4–10 years [10]. Shorter sleep duration, especially in the presence of irregular sleep patterns, was associated with higher plasma fasting insulin, low-density lipoprotein cholesterol (LDL-C), and C-reactive protein concentrations [12]. Fatma Kubra Sayin, noted a U-shape association between sleep duration and metabolic risk factors, with decreased HDL-C and decreased LDL-C and TG at lower sleep durations [12]. It is possible that the short sleep may lead to the dysregulation of adipokine secretion and is thought to be the key events in promoting both systemic metabolic dysfunction and cardiovascular disease. The change of sleep structure and the shortening of sleep duration can change many factors related to blood lipid regulation, such as leptin, adiponectin, FGF, RBP4 and so on [13]. Lipid metabolism is regulated by the circadian system, and the expression of enzymes involved in lipid metabolism is directly controlled by the circadian clock genes [14]. We speculated the following explanations for the positive association between the severity of SDB and LDL-C, HDL-C and LDL/HDL. And in the none-obese group we also found the abnormal TC, HDL-C and LDL/HDL have relation with SDB. Chronic sympathetic nervous activation caused by SDB even PS could increase the synthesis of very low-density lipoprotein in the liver and suppress the catabolism of LDL in the liver by stimulating α1-receptors, leading to a decrease in serum HDL-C and an increase in serum LDL-C and thereby an increase in LDL-C/HDL-C [9].

In the present study, L-SpO2 was independently associated with TG. Hepatic steatosis reversibly decreases viability of hepatocytes after hypoxia and reoxygenation in vitro [15]. It is hypothesized that in obesity, deregulation of inflammatory adipocytes is a response to the hypoxia that develops in adipocytes that are distant from the vasculature as adipose tissue expands; hypoxia directs the inflammatory response to adipose tissue, which includes new formation of veins [16]. This response to hypoxia is mediated by the transcription factor HIF-1a, the activity of which is now considered to indicate hypoxia in adipose tissue. Therefore, hypoxia may be one of the mechanisms leading to abnormal TG and obese [17]. In addition, sleep curtailment is associated with hormonal changes including increased cortisol and ghrelin levels, sympathovagal responses and reduced leptin [18]. Persistent insulin resistance or the presence of SDB somehow triggers weight gain. It is generally found that sleep and obesity are important factors affecting serum lipid metabolism in children. Li found obese children have significantly lower circulating plasma adiponectin and higher leptin levels than their nonobese counterparts, irrespective of their OSA status [18]. The BMI-z but not OSA was an independent predictor for both adiponectin and leptin. It is common knowledge that TG obese children increase. The study of Verhulst,et al. in subjects who were overweight and obese showed that the severity of SDB was independently correlated with a few factors associated with metabolic dysregulation [19]. Obesity is also a strong risk factor for adult SDB. Although data in children are inconclusive, obesity has been described as a risk factor for snoring or SDB in samples of older children and adolescents [20]. Our data are consistent with this and underscore the importance of overweight as an SDB risk factor in adolescents.

We observed that male gender and short sleep duration were associated with obesity. Although gender is a non-modifiable traditional cardiovascular risk factor, this underscores the identification of male subjects for more detailed evaluation and education on healthy sleep habits [21]. 

In aggregate, these findings point to SDB, as a contributing cause of dyslipidemia in SDB, and suggest the need for further research aimed both at elucidating the potential causal pathways that may link SDB and metabolic dysfunction, and whether treatment of SDB improves metabolic dysfunction in children.

## Conclusion

SDB has a very obvious effect on blood lipids, whereas PS without apnea and hypoxia at night also has an adverse impact on children’s blood lipids metabolism. L-SpO2 maybe only affect TG, but have no significant impact on TC, HDL-C, LDL-C and LDL/HDL. Obese (BMI-Z) only affects the aggregation of TG and does not significantly affect other blood lipid indicators. Our data may provide some public health information and provide new insights into the relationships between lifestyle habit and health. These form the rationale for the development of interventional strategies and educational programs to promote healthy sleep habits in the young population.

### Limitation

We did not have PSG results of control group for comparison. Because it is difficult to subject asymptomatic children (control group) to an overnight sleep study. Through epidemiological and clinical research, we can analyze the relationship between children’s dyslipidemia and sleep, but as for the specific pathogenic factors and the close degree of correlation, we need to conduct genetic studies and analysis. Further studies are needed to investigate the pathogenetic mechanisms in exploring the causal relationship between sleep, obesity and lipid metabolism.

## Data Availability

The datasets used and/or analysed during the current study are available from the corresponding author on reasonable request.

## Abbreviations

SDB: Sleep-disordered breathing
PS: Primary snoring
OSAS: Obstructive sleep apnea syndrome
TC: Total cholesterol, TG:triglyceride
HDL-C: High-density lipoprotein cholesterol
LDL-C: Low-density lipoprotein cholesterol
L-SpO2: Lowest oxygen saturation
M-SpO2: Mean oxygen saturation
BMI: Body mass index
CDC: Centers for Disease Control
AHI: Apnea/Hypopnea Index
ORI: Oxygen reduction index
L-SpO2: Lowest oxygen saturation, M-SpO2:mean oxygen saturation
